# Editorial: When the drug induces kidney diseases: nephrotoxicity and intoxication/poisoning

**DOI:** 10.3389/fmed.2025.1615283

**Published:** 2025-05-19

**Authors:** Welder Zamoner, Rodrigo Bueno de Oliveira, Etienne Macedo

**Affiliations:** ^1^Division of Nephrology, Department of Internal Medicine, Botucatu School of Medicine, University of São Paulo State-UNESP, Botucatu, Brazil; ^2^Division of Nephrology, Department of Internal Medicine, School of Medical Sciences, University of Campinas-UNICAMP, Campinas, Brazil; ^3^Division of Nephrology and Hypertension, Department of Medicine, University of California, San Diego, San Diego, CA, United States

**Keywords:** kidney disease, drug, nephrotoxicity, acute kidney injury, poisoning

This Research Topic of *Frontiers in Medicine* highlights the growing burden of drug-induced acute kidney injury (DI-AKI), a multifaceted and increasingly relevant clinical problem. The articles featured explore various dimensions of DI-AKI, ranging from nephrotoxicity in oncology to evolving diagnostic tools, epidemiology, and therapeutic strategies.

Garcia et al. conducted an observational study of 1,398 patients with AKI, showing that ~20% of cases were associated with nephrotoxic exposures, predominantly vancomycin, iodinated contrast, and aminoglycosides. While these DI-AKI cases demonstrated lower mortality compared to other AKI etiologies, the need for acute kidney replacement therapy (AKRT) was similar. These findings support growing interest in the development of bundle care strategies for DI-AKI that may mitigate the severity and long-term impact of these events. For example, as discussed by Colares et al., even in patients with normal serum creatinine, a simple bundle care such as serum vancomycin concentration monitoring may allow appropriate adjusts to avoid DI-AKI development (1). Chegini et al. examined the impact of methadone syrup in a cohort of 150 individuals undergoing maintenance therapy. Their findings suggested a possible dose-related decline in eGFR, though design limitations precluded firm conclusions. Nevertheless, this study underscores the need for more rigorous evaluation of understudied nephrotoxic exposures, especially given the rising prevalence of opioid use.

Zhou et al. provide a timely review of the renal complications of immune checkpoint inhibitors (ICIs). These agents, now standard therapies in multiple malignancies, have been increasingly implicated in AKI, predominantly manifesting as acute tubulointerstitial nephritis (ATIN). Mechanistic insights include T-cell activation, loss of peripheral tolerance, and cross-reactivity with renal antigens. The nephrotoxicity of ICIs may be potentiated when used in combination regimens, such as anti-CTLA-4 plus anti-PD-1. As discussed by Casals et al. (2), recent studies have proposed immunohistochemical markers such as PD-1 and PD-L1 staining in renal biopsies to differentiate ICI-related AIN from other causes. This may be particularly useful when patients are on multiple concurrent nephrotoxic agents.

In their case series, Tome et al. presented patients with methotrexate-related AKI and highlighted the role of high-flux hemodialysis and glucarpidase in managing severe toxicity. These findings align with data discussed in the 2024 *AJKD Core Curriculum* by Krishnan et al. (3), which reviews drug-induced crystalline nephropathies, a classic yet underrecognized mechanism of DI-AKI involving agents such as methotrexate, sulfadiazine, acyclovir, and indinavir.

Recent studies emphasize the need for standardized clinical and histopathologic criteria and suggest that early diagnosis and minimal fibrosis are associated with better outcomes (4, 5). Noninvasive diagnostic tools are under investigation, including gallium-67 scintigraphy and the lymphocyte transformation test (LTT), which may help identify AIN and its causative agents, particularly in patients on immunotherapy (6, 7). Biomarkers are also gaining traction. Moledina and colleagues (5) identified urinary cytokines, particularly TNF-α and IL-9, as promising diagnostic markers for AIN in a prospective study. These markers outperformed clinical suspicion and standard laboratory parameters in distinguishing AIN from acute tubular injury (ATI) and other AKI subtypes.

The evidence presented across this Research Topic reinforces the need for comprehensive epidemiological studies that address the heterogeneous phenotypes and outcomes of DI-AKI. There is increasing momentum behind the development of biomarker-based diagnostics, which may help circumvent the limitations of kidney biopsy. The role and timing of corticosteroids remain controversial, but studies such as that by Miao et al. suggest early initiation may improve outcomes, especially in drug-induced AIN. There is also a call, as articulated by Moledina and Perazella (5), for expert consensus or guideline development on the selection, dosing, and duration of immunosuppressive therapy for AIN.

In a rapidly evolving therapeutic landscape shaped by new cancer treatments, complex polypharmacy, and an aging population, DI-AKI remains a critical frontier for both clinical care and translational research. The path forward demands multidisciplinary collaboration to refine risk stratification, improve diagnostic accuracy, and define treatment paradigms that preserve kidney function without compromising necessary therapy.
